# Parameters as Trait Indicators: Exploring a Complementary Neurocomputational Approach to Conceptualizing and Measuring Trait Differences in Emotional Intelligence

**DOI:** 10.3389/fpsyg.2019.00848

**Published:** 2019-04-18

**Authors:** Ryan Smith, Anna Alkozei, William D. S. Killgore

**Affiliations:** ^1^ Laureate Institute for Brain Research, Tulsa, OK, United States; ^2^ Department of Psychiatry, University of Arizona, Tucson, AZ, United States

**Keywords:** trait emotional intelligence, computational neuroscience, reinforcement learning, Bayesian brain, computational modeling, assessment

## Abstract

Current assessments of trait emotional intelligence (EI) rely on self-report inventories. While this approach has seen considerable success, a complementary approach allowing objective assessment of EI-relevant traits would provide some potential advantages. Among others, one potential advantage is that it would aid in emerging efforts to assess the brain basis of trait EI, where self-reported competency levels do not always match real-world behavior. In this paper, we review recent experimental paradigms in computational cognitive neuroscience (CCN), which allow behavioral estimates of individual differences in range of parameter values within computational models of neurocognitive processes. Based on this review, we illustrate how several of these parameters appear to correspond well to EI-relevant traits (i.e., differences in mood stability, stress vulnerability, self-control, and flexibility, among others). In contrast, although estimated objectively, these parameters do not correspond well to the optimal performance abilities assessed within competing “ability models” of EI. We suggest that adapting this approach from CCN—by treating parameter value estimates as objective trait EI measures—could (1) provide novel research directions, (2) aid in characterizing the neural basis of trait EI, and (3) offer a promising complementary assessment method.

## Introduction

The trait model of emotional intelligence (EI) conceptualizes EI as a set of self-perceived competencies, such as high adaptability, high stress tolerance, high optimism, and low impulsiveness, among others (Pérez et al., 2005; Petrides et al., 2016). In this approach, self-report inventories are used that ask people to evaluate their own attributes in this domain, similar to the self-report inventories used to assess personality variables (e.g., Austin et al., 2004). Many measures of EI have been developed within this model, with the Bar-On Emotional Quotient Inventory (EQ-i) and the Trait Emotional Intelligence Questionnaire (TEIQue) representing two prominent examples (e.g., see Bar-On, 2004; Mikolajczak et al., 2007).

Higher trait EI scores on such measures have previously been associated with better social adjustment (Engelberg and Sjöberg, 2004), better recovery from traumatic experience (Hunt and Evans, 2004), a stronger attentional bias toward positive affective stimuli (Lea et al., 2018), higher levels of extraversion, conscientiousness, openness, and agreeableness, and lower levels of neuroticism (Warwick and Nettelbeck, 2004; van der Linden et al., 2017), as well as with a generally more positive mood (Schutte et al., 2002; but see Spence et al., 2004). Higher trait EI scores have also been linked to lower work-related stress (Dulewicz et al., 2003), a lower likelihood of reporting symptoms diagnostic of some psychological disorders (Hemmati et al., 2004), and lower symptom severity in clinical samples (Petrides et al., 2017).

While the aforementioned findings were based on associations with other self-report measures, significant relationships have also been found with a few objective measures. For example, those with higher self-reported trait EI show faster facial emotion recognition (Austin, 2004), as well as better job performance and better indices of health (Martins et al., 2010; O’Boyle et al., 2011), than those scoring lower on this trait. A few previous studies using functional magnetic resonance imaging (fMRI) have also shown that trait EI scores are correlated with functional and/or structural differences in the prefrontal cortex, insula, and amygdala (Killgore and Yurgelun-Todd, 2007; Takeuchi et al., 2011, 2013; Killgore et al., 2012, 2013; Smith et al., 2016). This growing body of work supports the validity and importance of the trait EI construct.

One theoretical difficulty in using this subjective-rating approach to measure trait EI, however, is that individuals’ self-evaluations can be inaccurate. For example, the relationships observed between trait and performance-based or third-party observer measures of EI tend to be weak (e.g., r = 0.20 to 0.30; Carney and Harrigan, 2003; Brackett et al., 2006; Goldenberg et al., 2006; Brannick et al., 2009; Webb et al., 2013). Other work has also found, for example, that people who rate their own social sensitivity highly receive much lower ratings from third-party observers (Carney and Harrigan, 2003). Another concern is that associations between trait EI scores and self-reported personality measures are quite robust (Warwick and Nettelbeck, 2004; De Raad, 2005), and recent suggestions have been made that trait EI scores might primarily reflect a general latent personality factor associated with lower neuroticism and higher extraversion, openness, conscientiousness, and agreeableness (van der Linden et al., 2017). On one hand, this link between trait EI and basic personality dimensions may provide support for its biological basis, as the observed correlations appear to be due (in part) to shared genetic and neuroscientific underpinnings (Vernon et al., 2008; Mikolajczak et al., 2010; Petrides et al., 2016). On the other hand, however, if the trait EI construct is intended to track true individual differences in social/emotional competencies over and above those measured by broad personality variables, one might not expect such differences to be so strongly associated with those personality variables; this also raises the concern that these strong associations could in part reflect shared self-report variance. These issues highlight the potential usefulness of more objective measures of an individual’s traits.

This is especially true in the context of the increasing neuroscience research on trait EI (and on the potential to use neural/biological measures as markers of improvements in EI; Pérez-González and Qualter, 2018) referred to above, as certain theoretical difficulties arise in correctly interpreting such neuroimaging results. For example, when differences in neural responses are associated with objective performance differences, these neural responses are typically interpretable as reflecting underlying differences in relevant neural processes (Smith et al., 2018). For instance, in the distinct “ability model” of EI (Mayer et al., 2003), which instead conceptualizes EI as a set of abilities assessed by task performance (e.g., the ability to recognize and regulate emotions), brain activity associated with better task performance can be plausibly linked to processes contributing to the use of those abilities. The objective nature of the ability model has been one of its most valuable features, which has led to its common use in EI research and its conceptualization as similar to other traditional metrics of cognitive intelligence (Mayer et al., 2001). (Note: Drawbacks to this approach have also been highlighted, however (Roberts et al., 2001, 2006; Matthews et al., 2007; Fiori et al., 2014; Smith et al., 2018); for example, many ability EI measures use consensus scoring (as opposed to having objective accuracy metrics) and therefore cannot include difficult items that could appropriately measure above average levels of EI.).

In contrast, trait EI scores most directly reflect individuals’ *beliefs* (self-assessments or self-perceptions) about themselves, and those beliefs need not co-vary highly with objective assessment (beliefs about oneself can nonetheless have important influences on brain and behavior, however; e.g., see Smith et al., 2016). In addition, subjective ratings of trait EI are often understood to reflect *typical* (or habitual) performance in real-world situations as opposed to the *optimal* (i.e., highly motivated, goal-directed) performance assessed in laboratory tasks and other objective performance measures of EI-related abilities. As such, interpreting the meaning of neuroimaging findings regarding trait EI can be less straightforward because the neural basis of subjective beliefs and of typical performance are likely different than the neural basis of optimal performance assessed objectively (for a recent example attempt to interpret distinct neural correlates of trait and performance measures of EI, see Smith et al., 2016). Further research in the field of trait EI could be enhanced, therefore, by developing methods to measure such traits with more objective metrics.

Based on these considerations, the present paper explores one possible way in which EI-relevant traits could be assessed objectively, derived from the field of computational cognitive neuroscience (CCN). Briefly, we present the thesis that the notion of “model parameters” in CCN represents a novel way to think about psychological traits in the field of EI research. As we will describe below, these parameters can be estimated for individual participants in objective behavioral tasks and therefore do not rely on self-report. Yet, they reflect stable individual differences with a different character than the performance abilities assessed by ability EI models. Thus, they provide a potential framework in which trait-like factors, that have more easily interpretable neural underpinnings, can be assessed in an objective manner. This builds on previous work that has suggested ways in which neuroimaging measures could also be used to more objectively assess differences in EI-relevant traits (e.g., assessing more or less adaptive “affective styles” based on differences in asymmetric prefrontal activation and amygdala responses; Davidson and Irwin, 1999; Davidson, 2004; Davidson and Begley, 2012).

In the following sections, we will expand upon the thesis that computational neuroscience measures can be used to assess socio-emotional traits and then provide several examples of specific model parameters that have been derived in recent research, which appear to overlap with, or at least be highly relevant to, the emotional competencies associated with trait EI. Ultimately, we will argue that expanding on this approach within the field of EI research could represent a promising avenue for future studies.

## Computational Cognitive Neuroscience

The field of CCN starts with the premise that neural networks within the brain implement information processing mechanisms that can be described by mathematical equations. It then focuses on creating and testing mathematical models and their ability to account for human behavior and brain activity. There is a wide range of different types of models used within this field, many working at different levels of description. For example, some of this work has focused primarily on the use of mathematical models of cognition (typically based on Bayes’ theorem in probability theory), with little emphasis on underlying neural implementation (e.g., see Kemp et al., 2007; Xu and Tenenbaum, 2007a,b; Perfors et al., 2011). Other work has focused on algorithms capable of learning to behave optimally in the context of seeking reward and avoiding punishment, which has included considerable focus on brain mechanisms (Sutton and Barto, 1998; Frank, 2011; Dolan and Dayan, 2013; Gershman, 2017). Finally, there is a growing body of work on what has been called the “free-energy principle” (Friston, 2010), which has provided concrete neural models (at the level of interconnected neurons and their dynamics) of perception/attention (Friston, 2005; Kiebel et al., 2008; Feldman and Friston, 2010; Parr and Friston, 2017), learning and decision-making (Friston et al., 2016, 2017), emotion and visceral regulation (Pezzulo et al., 2015; Seth and Friston, 2016; Stephan et al., 2016; Smith et al., 2017; Owens et al., 2018), and skeletomotor control (Friston et al., 2010).

According to these CCN models, the brain must store and use stable values for a range of parameters (e.g., expected levels of reward in a particular situation, prior expectations about what is most likely to be perceived, etc.). Some of the parameter values stored in the brain may be inherited, while others are plausibly learned from experience (perhaps early experience especially), and they need not be identical across individuals. Further, although many may be slowly altered through further learning, such parameters can often be treated as stable individual differences. Broadly speaking, by storing and using specific values for a range of different parameters, the brain is thought of as implementing an “internal model” of the world that an individual uses to guide perception, bodily regulation, and behavior.

Of primary relevance to the present paper, multiple parameter values within an individual’s internal model may relate in important ways to trait differences in EI-relevant competencies. Further, behavioral paradigms have been created that can estimate these parameters for individual participants, providing useful trait-like information. In what follows, we will review previous work in which such experimental paradigms have been used to estimate individual differences in some specific parameter values, and we will demonstrate why these are more plausibly considered traits than abilities. We will then illustrate how mapping trait differences in these parameter values to trait differences in EI may provide conceptual and empirical tools capable of advancing research on trait EI. To be clear, the work we will review does not offer a complete list of EI-relevant parameters. Our goal is instead to demonstrate how such an approach could be expanded to assess other EI-relevant traits in a novel manner.

## Specific Example Parameters Relevant to Trait EI

### Mood Stability

The first parameter we will consider corresponds to trait differences in mood stability, based on a recent body of work within the literature on reward learning (Eldar and Niv, 2015; Eldar et al., 2016, 2018; Mason et al., 2017). Briefly, the computational models used in this work highlight an important interaction between mood stability and the way events involving reward or loss are processed. Specifically, this work has shown that (1) mood is improved after repeatedly experiencing greater reward than expected, and (2) outcomes are perceived as more rewarding when a person is in a good mood relative to when they are in a bad mood. In symmetric fashion, losses are perceived as worse when someone is in a bad mood, and the repeated experience of unexpected loss further worsens mood. This two-way interaction may be helpful in improving the efficiency of reward learning in certain environments, but it also creates the potential for positive feedback loops that can lead to mood instability.

In the mathematical equations used by Eldar and colleagues to model these processes, one particular parameter (*f*) controls the strength of the effect that mood has on perception of subsequent outcomes (i.e., higher *f*-values lead good mood to more strongly amplify perceived reward and bad mood to more strongly amplify perceived losses). Using a behavioral task combining mood induction and reward learning, they were able to estimate the best-fit values of *f* for each participant. They subsequently found that those participants with higher *f-*values were also more susceptible to mood instability (assessed using the hypomanic personality scale; Eckblad and Chapman, 1986) and that the associated amplified responses to reward corresponded to altered activation of striatal reward systems in the brain (Eldar and Niv, 2015). Briefly, this instability arises with higher *f-*values because reward (positive reward prediction-error) and mood amplify each other until expected rewards are unattainable, leading to losses (negative reward prediction-errors) and mood reductions that in turn amplify each other (and the cycle continues).

In this first example, the parameter value for *f*, although measured objectively, is not plausibly understood as a measure of task performance. Instead, it reflects a trait difference in vulnerability to mood instability that can be understood in mathematical and mechanistic neural terms. That is, it reflects the fact that positive mood amplifies perceived reward too much and negative mood amplifies perceived negative outcomes too much, corresponding to amplified neural responses in reward learning systems. Given that mood stability is an EI-relevant trait, this objective means of assessing this trait appears highly relevant. For example, *f* appears strongly related to the Emotion Regulation facet on the TEIQue and could also overlap somewhat with other TEIQue facets and factors, such as trait happiness, adaptability, well-being, and self-control. It could also relate to certain scales and subscales on the Bar-On EQ-i, such as adaptability, general mood, flexibility, and happiness. The potential relationships between *f* and the aforementioned EI traits have not yet been examined, representing one interesting direction for future research.

### Stress Vulnerability

The second parameter we will consider corresponds to trait differences in stress vulnerability. This is based on a recent study of uncertainty learning (de Berker et al., 2016), which linked differences in particular aspects of uncertainty-related behavior to differences in autonomic and endocrine responses associated with stress. In this study, individuals had to repeatedly guess the probability of receiving a shock after seeing various stimuli. Importantly, the probabilities shifted unpredictably over time. Based on behavior in this task, one of the parameters estimated by their computational model for each participant (*ϑ*) captures trait differences in expected levels of a specific type of uncertainty called “metavolatility.” Specifically, higher *ϑ*-values can be thought of as indexing stronger implicit expectations that the world is unstable (i.e., greater general uncertainty about the stability of learned relationships between events). Interestingly, they found that individuals with higher *ϑ*-values reported greater chronic life stress (assessed using the perceived stress scale; Cohen et al., 1983).

Individual differences in *ϑ* therefore reflect something like trait differences in vulnerability to stress (i.e., due to differences in implicit beliefs about world stability), and it also does not map onto any straightforward performance ability. This therefore provides another example of an objectively measureable individual difference variable relevant to trait EI. For example, *ϑ* appears strongly related to the stress management facet on the TEIQue and could also overlap somewhat with other TEIQue facets and factors, such as emotion regulation, trait happiness, adaptability, well-being, and self-control. It appears strongly related to the stress management scale and stress tolerance subscale on the EQ-i and could also overlap somewhat with other EQ-i scales/subscales, such as adaptability, general mood, flexibility, and happiness. Future research should examine whether these potential relationships exist.

### Optimism

The third parameter we will consider corresponds to trait differences in optimism, based on a recent study of expectation learning (Stankevicius et al., 2014). In this study, individuals were exposed to stimuli that were either followed by reward or no reward on each trial with a probability fixed for each stimulus (but differing for different stimuli), which was unknown to participants. After a few presentations of a given stimulus, participants were instructed to maximize reward by choosing either that stimulus or a novel stimulus with an explicitly indicated probability of reward. Using a mathematical model that assumed optimal probabilistic (Bayesian) inference, participants’ behavior could be used to estimate a set of beta-distribution parameters (α, β) that can be combined to measure each participant’s mean prior expectation of receiving future rewards (α/(α + β)). Subsequently, they found that (α/(α + β)) values were significantly positively correlated with trait optimism (assessed using the Life Orientation Test-Revised [LOT-R]; Hirsch et al., 2010), but that they did not correlate with several other personality variables.

Thus, this parameter represents an objective behavioral measure of trait differences in optimism, conceptualized computationally as stored prior expectations about the quantitative probability of receiving future rewards (i.e., likely related to brain regions involved in reward prediction, such as the anterior cingulate, basal ganglia, dopaminergic midbrain, and ventral/medial prefrontal cortex; Niv et al., 2007; Dolan and Dayan, 2013; Silvetti et al., 2014). This parameter appears strongly related to the trait optimism facet on the TEIQue and could also overlap somewhat with other TEIQue facets and factors, such as trait happiness, self-motivation, and well-being. It also appears strongly related to the optimism subscale on the EQ-i and could overlap somewhat with other EQ-i scales/subscales as well, such as adaptability, general mood, flexibility, and happiness. As with the other parameters already discussed, future trait EI research will be necessary to investigate these relationships.

### Flexibility and Self-Control

A fourth set of parameters we will consider correspond to overlapping traits that could be described as self-control, patience, flexibility, or goal-directedness. This broad idea has been assessed within reinforcement learning studies on “model-based” and “model-free” decision-making algorithms (Daw et al., 2005, 2011; Kool et al., 2016; Gershman, 2017). Briefly, model-based decision-making algorithms mathematically characterize a goal-directed process in which one consciously imagines a number of probable outcomes based on different action choices and then selects the action with the best expected outcome. This process is psychologically flexible, but computationally expensive and cognitively effortful. In contrast, model-free algorithms mathematically characterize a process in which habitual behaviors are learned based on statistical patterns of better and worse outcomes in past experience. Psychologically, one simply feels an impulse or automatic tendency to act a certain way in a given situation, because it has tended to lead to better outcomes in the past—although an individual need not be aware of this reason (e.g., similar to the implicit statistical learning observed in tasks such as the Iowa Gambling Task; Bechara et al., 1997; Gupta et al., 2011; Alkozei et al., 2018). Model-based and model-free algorithms appear to operate in a parallel and interactive fashion in the brain (e.g., model-based algorithms appear to primarily engage the dorsolateral prefrontal cortex and dorsomedial striatum, whereas model-free algorithms instead primarily engage ventral/lateral striatal regions), and it is thought that they compete for control of action (Daw et al., 2005). Most relevant to the present discussion, decision-making tasks (Kool et al., 2016) have been developed that assess quantitative individual differences in (i.e., parameter values describing) the default tendency to engage in model-based (flexible, goal-directed) or model-free (rigid, impulsive) decision making. Those that have higher “model-based” parameter values will therefore tend to be more flexible and goal-directed and less impulsive. It is worth emphasizing that these parameter values do not necessarily reflect a person’s *ability* to engage in the controlled/deliberative type of cognition associated with model-based algorithms; instead, they are better understood to reflect the degree to which an individual will *typically* engage in this type of cognition when it would be beneficial.

A related set of parameters has also been assessed within neural “active inference” models that are based on the free-energy principle (i.e., according to this principle, individuals act so as to minimize an information-theoretic quantity related to surprise; Schwartenbeck et al., 2015; Schwartenbeck and Friston, 2016; Parr and Friston, 2017; Friston et al., 2018). One parameter, called *policy precision*, encodes an individual’s *a priori* confidence that one action option will be better than others; this parameter is linked to dopaminergic signaling in these models (Parr and Friston, 2018). Higher policy precision values entail that behavior is more deterministic (i.e., less random). A second parameter in these models, called *transition precision*, encodes an individual’s a priori confidence in the predictability of future events; this parameter is linked to noradrenergic signaling in active inference models (Parr and Friston, 2018). Higher transition precision values indicate the implicit belief that distant future states are more predictable, entailing more goal-directed (i.e., more patient, less impulsive) decision-making strategies (e.g., being willing to forego smaller rewards now to receive larger rewards later—which only makes sense if the more distant rewards are predictable enough to “bet on”; Mirza et al., 2019). Different combinations of policy and transition precision values can therefore characterize an individual’s tendencies toward acting randomly and impulsively vs. acting in a more patient and controlled manner.

As such, these overlapping parameters appear strongly related to the (low) impulsiveness facet, self-motivation facet, and self-control factor on the TEIQue and could also overlap somewhat with the adaptability facet. They also appear strongly related to the impulse control, flexibility, and problem solving subscales on the EQ-i and could also overlap somewhat with the broader adaptability scale. In addition, given that some other self-reported EI skills (e.g., regulating/managing the emotions of self and others) make use of controlled/effortful cognition, those with greater tendencies to engage in these processes (as assessed by the aforementioned parameter estimates) might appear to show greater skills levels in typical situations. While these parameters appear to be similar to the constructs measured by traditional self-report measures of trait EI, these associations remain to be tested through empirical research.

### The Influence of Automatic Affective Action Tendencies

The fifth parameter we will consider corresponds to trait differences in the influence of affective valence on behavior, based on recent work in reinforcement learning (Guitart-Masip et al., 2012, 2014). Briefly, this work has illustrated interactions between the expected valence (pleasantness or unpleasantness) of outcomes and behavioral tendencies when trying to achieve those outcomes (i.e., which may correspond to interactions between brain regions encoding value/valence (e.g., ventral/medial prefrontal cortex) and regions controlling action (e.g., striatum); see Guitart-Masip et al., 2014). More specifically, expected pleasant outcomes appear to promote Pavlovian (biologically pre-specified) approach behaviors, whereas expected unpleasant outcomes promote Pavlovian inhibition or avoidance behaviors. This can lead to suboptimal responses in situations where, for example, long-term success requires approaching situations to avoid unpleasant outcomes (e.g., requiring inhibition of biologically prepared inhibition/escape tendencies).

Importantly, individuals differ in the degree to which expected valence influences their behavior in this manner. One task designed to assess this interaction between valence and behavior is the orthogonalized go/no-go task (Crockett et al., 2009). In this task, individuals either have to act or inhibit an action (go and no-go, respectively) to either win or to avoid losing something of value (i.e., four combinations, each in a different task condition). In this task, some individuals perform worse than others, with varying levels of impaired learning/performance in the “go to avoid losing” and “no-go to win” conditions (i.e., relative to the other two conditions where valence and automatic behavior agree; e.g., see Guitart-Masip et al., 2012; Chowdhury et al., 2013). Individual differences in the ability to learn/perform in these conditions where valence and action disagree (i.e., individual differences in parameters describing the strength of the interaction between valence and action) have been used as measures of psychological flexibility (e.g., to examine individual differences in flexibility between younger and older adults; Chowdhury et al., 2013).

There is a range of circumstances in which this could pertain to EI-relevant traits. For example, there are social circumstances in which one must approach uncomfortable social and workplace situations to avoid even more unpleasant long-term outcomes. More generally, this objectively measurable trait difference could theoretically correspond closely with the (low) impulsiveness facet, emotion regulation facet, and self-control factor on the TEIQue and could also overlap somewhat with other TEIQue facets, such as self-motivation and adaptability. It also appears strongly related to the flexibility and impulse control subscales on the EQ-i and could also overlap somewhat with its broader adaptability scale. Yet, no study to date has attempted to link trait EI scores to this objective measure of behavioral flexibility in affective contexts.

### Cognitive Flexibility in Reality Testing

The final parameter we will consider corresponds to trait differences in the degree to which people are cognitively flexible and test their beliefs before acting on them. In decision-making research, this has been studied in the context of the “explore/exploit dilemma” (Sutton and Barto, 1998; Berger-Tal et al., 2014), which refers to the difficult problem of deciding when to trust (and act on) a previously learned model of the world (“exploiting”) and when to first check (before acting) to make sure that model of the world is still accurate (“exploring”). In neuroscience research, evidence suggests that both noradrenergic and dopaminergic signaling play a role in modulating how (and how flexibly) individuals solve this problem (Aston-Jones and Cohen, 2005; Beeler et al., 2014).

Solving this problem adaptively is highly relevant to psychopathology and its treatment (Addicott et al., 2017). For example, individuals with early adversity may learn a maladaptive model of social interactions (e.g., “people will always hurt me and take advantage of me if I show emotional vulnerability”) that, while true of their childhood environment, is not true of their broader social environment in adulthood. Similarly, individuals with mood and anxiety disorders often have acquired maladaptive socio-emotional beliefs (e.g., “no one would want to be my friend”). In both cases, habitually acting on such beliefs can prevent the ability to learn a more adaptive model; for example, by socially isolating one’s self or being preemptively cold and defensive during social interactions, one can inadvertently elicit reactions from others that maintain those maladaptive beliefs. In both cases, evidence-based psychotherapeutic interventions (Hayes and Smith, 2005; Barlow et al., 2016)—and exposure techniques in particular—also focus on countering maladaptive cognitive/behavioral habits by promoting more flexible, exploration-based cognition and behavior as a way of promoting more adaptive learning (e.g., “let yourself show some emotional vulnerability and see if it goes the way you expect”).

There are a number of tasks within the reinforcement learning literature that have been used to assess this type of flexible reality testing (reviewed in Addicott et al., 2017). As one illustrative example, a task called the “horizon task” asks participants to make a series of choices between two slot machines with the goal of maximizing earnings, after having seen different numbers of examples of previous payouts from each of the two machines (Wilson et al., 2014). To estimate trait differences in a “goal-directed exploration” parameter, participants’ early choices in this task can be examined when they are given more vs. less information about previous payouts from one slot machine or the other. Interestingly, while individuals high in goal-directed exploration tend to first “test out” the slot machine that they know less about, those lower in this trait tend to just pick the one with higher past payouts and stick to it (i.e., regardless of how many past payouts they have seen). Thus, those with higher goal-directed exploration parameter values appear more sensitive to uncertainty and try to gather more information before “jumping to a conclusion” too quickly—often leading to better overall performance.

Although primarily studied thus far in the context of simple gambling tasks, trait differences in goal-directed exploration during decision-making are of clear relevance to intelligent, adaptive, and flexible social and emotional responding. Conceptually, this trait difference appears related to the (low) impulsiveness facet and adaptability facet of the TEIQue. It also appears strongly related to the reality testing subscale of the EQ-i, as well as other components of its adaptability scale (i.e., flexibility and impulse control). However, no study to date has attempted to link trait EI scores to this objective measure of flexibility and reality testing; adapting such tasks to study goal-directed exploration in explicitly socioemotional contexts is also an important future research direction.

## A Broader Perspective on Model Parameters and Trait EI

In the previous section, we provided several specific examples of parameter values that can be experimentally estimated for individuals and that could plausibly relate to trait differences in a range of EI-related competencies. We also demonstrated why they correspond much more closely to the notion of traits than to the notion of specific task performance abilities (or to typical as opposed to optimal behavior). However, the examples provided above certainly do not provide comprehensive coverage of EI-relevant traits. For example, they do not appear to correspond directly to traits such as self-esteem, assertiveness, emotional awareness, interpersonal relationship competencies, among others (i.e., although they could certainly have indirect influences on these other traits; e.g., optimism parameters could promote self-esteem, and parameters related to patience, self-control, cognitive flexibility, and low impulsiveness could aid in interpersonal relationships, etc.). Given this lack of complete coverage, our claim is not that the tasks described above could exhaustively assess trait EI in an objective manner. Our claim is instead that these examples from CCN may provide a blueprint for ways in which one could design tasks to assess other trait EI facets, and that doing so could both avoid potential issues linked to self-report and also facilitate neuroscience research on trait EI. The resources for doing so can also be further clarified by giving broader consideration toward other general parameters that must be stored unconsciously within any plausibly human mind. In fact, there are some general categories of parameter estimates that are necessary for perception and action, which have clear relevance to EI competencies. We consider a few below.

### Prior Probability and Precision Estimates

The sensory input received by the brain is known to be noisy and ambiguous. Put another way, there are always multiple possible causes of the same sensory input. As such, the brain must be equipped with (and be capable of updating) prior expectations (i.e., stored probability values typically referred to as “priors”) about which causes are more likely than others in general and use these expectations to infer the most likely cause of any particular pattern of sensory input (i.e., similar to the example of optimism and prior expectations of future reward above). As there are a vast number of possible causes out in the world that interact in complex ways, this entails that the brain must store a large number of priors. This is the general basis for a large number of current probabilistic (Bayesian) models of brain function (Knill and Pouget, 2004; Friston, 2010). Not all of these priors may relate to trait EI; however, several plausibly do (e.g., for specific theoretical applications to social perception/behavior, see Diaconescu et al., 2014; Friston and Frith, 2015; Sevgi et al., 2016).

As one example, consider a woman who observes a man with a neutral facial expression. As such expressions are more or less consistent with a range of emotional states, prior expectations will play an important role in emotion recognition competencies (e.g., expectations about what facial features correspond to what emotions, about what emotions are more likely in general, and about what emotions are more likely in a particular situation). For instance, if the woman has a prior expectation that people typically tend to feel happy, then—all else being equal—the woman would perhaps perceive the man’s neutral expression as indicating mild happiness. In contrast, a different prior expectation could promote the perception of a range of other emotions. Prior expectations can also be specific to context. For example, assume the man is observed at a family gathering. If the woman has a prior expectation that family gatherings are generally enjoyable, then she would likely perceive the man as happy; in contrast, if she expects that family gatherings are often awkward and tense, the man might instead be perceived as anxious or annoyed.

Individuals with prior expectations that are better calibrated to their social/cultural environment would therefore be expected to have higher competence in the domain of emotion perception. More generally, for most trait EI competencies, there will be similarly relevant priors. For example, individuals will have learned prior expectations for (1) their value in the eyes of others (self-esteem), (2) their ability to navigate the environment effectively (independence), (3) the probable mental states of others in specific situations and the types of actions that tend to make individuals feel better or worse (emotion management, social awareness, and empathy), and many others.

Competence within the perceptual and cognitive domains also requires estimating the reliability of particular sensory inputs and expectations (Feldman and Friston, 2010). For example, in the context of a sunny day, visual input should be treated as reliable and have a strong influence on perception and learning within the brain. In contrast, input from vision in the dark of night is much less reliable, and the brain could arrive at many false percepts if it was treated as though it were trustworthy. Thus, in Bayesian models of the brain, it is assumed that the brain learns context-specific estimates of when to put greater trust in sensory input and when to put greater trust in prior expectations. These reliability estimates correspond to a set of internal model parameters called “precision estimates,” which guide attention, belief updating, and decision-making in a context-specific manner. In fact, the policy and transition precision parameters discussed in section 3.4 specifically represent two such precision estimates, reflecting the reliability of predictions about sensory input based on chosen actions and beliefs about the past/future, respectively (for discussion of mood biases as reflecting different types of precision estimates, see Clark et al., 2018).

More generally, however, precision estimates can be seen as conveying a person’s confidence in a wide range of variables. For example, if one assigned a very strong precision estimate to their current beliefs, they would have great difficulty learning from new experience and would be unlikely to engage in the type of reality testing competency assessed in the EQ-i, or if an individual assigned very low precision estimates to social information in sensory input (e.g., effectively ignoring the facial expressions, body postures, and voice tones of others), they would be expected to have low social awareness. The structure of these examples is widely generalizable to other EI traits. Regardless of the specific example, however, the key task for expanding this approach to trait EI research would be to identify the relevant prior expectation and precision estimate parameters and then design tasks to quantitatively measure individual differences in these parameters *via* individuals’ behavior.

### Learned Action Values

The final example we will provide returns to the work on model-free decision algorithms discussed on section 3.4. In that section, we focused on the competition between these (habit-driven) algorithms and model-based (goal-directed) algorithms and did not discuss *what* habitual behaviors had been learned. However, competencies within the social/emotional domain also likely depend on the specific behavioral tendencies that an individual has learned through reinforcement in EI-relevant contexts. Within model-free algorithms, if a particular action (in a particular situation) has been repeatedly and reliably followed by positive outcomes, then the stored value of that action will be high for that situation; symmetrically, those actions typically followed by negative outcomes in personal experience with a specific situation will be assigned low values (Gershman, 2017). When again in that situation, model-free algorithms would select whatever action has the highest stored value, often corresponding to a strong subjective urge (or automatic tendency) to behave in a particular way (and strong urges to avoid choosing low-value actions—without an individual necessarily understanding why).

The important point here is that different stored action values can promote more or less emotionally/socially competent behavioral tendencies. Less adaptive behavioral tendencies can arise, for example, if they were learned in one environment but then applied in a different environment. For example, if assertive behaviors were reliably punished during childhood, an individual would likely have automatic tendencies toward passive (nonassertive) behaviors in adult social environments—even if assertive behaviors would in fact be rewarded in the latter environment. Importantly, because such an individual would have a strong aversion to “trying out” assertive behaviors (i.e., because their stored action values are so low), they may never have an opportunity to learn that such behaviors would now be followed by desirable outcomes (i.e., this is an example of failing to adaptively solve the explore/exploit dilemma described in the “Cognitive Flexibility in Reality Testing” section). A similar analysis of other behavioral facets of trait EI could also be provided, such as facets pertaining to socially responsible behavior, behavior in interpersonal relationships, and empathic behavior (among others). The major point is that a particular set of model-free parameters that promote selection of habitual actions (technically called “state-action pair values” or “Q-values”) could in principle be used as objective indices of some trait EI competencies. This would require that tasks were designed to estimate an individual’s stored values for different actions in EI-relevant situations, which has not been attempted to date.

## Conclusion

In this paper, we have discussed the idea that specific individual difference parameters estimated in CCN studies could be understood as objective assessments of traits, and that many do not appear to correspond as directly to performance abilities. We have also provided specific examples of parameters that appear to correspond to EI-relevant traits to various degrees and illustrated how more general theoretical resources within CCN could guide the development of tasks capable of assessing trait EI facets more comprehensively. This offers the possibility of a complementary means of assessing trait EI competencies that does not rely on self-report and which could perhaps better dissociate them from other self-report measures. Finally, we have highlighted that, because each of the parameters discussed above have previously examined neuroscientific bases, this should help extend recent work attempting to examine the neural basis of individual differences in trait EI. We contend that this approach therefore represents a promising complementary assessment approach for future emotional intelligence research.

## Author Contributions

RS took the lead in the conceptualization and writing of the manuscript. AA and WK aided in developing ideas, providing feedback, editing, and revising the manuscript.

### Conflict of Interest Statement

The authors declare that the research was conducted in the absence of any commercial or financial relationships that could be construed as a potential conflict of interest.
